# Effects of minimalist shoes on pelvic floor activity in nulliparous women during running at different velocities: a randomized cross-over clinical trial

**DOI:** 10.1038/s41598-022-25344-6

**Published:** 2022-12-08

**Authors:** María García-Arrabe, Pablo García-Fernández, Beatriz Ruiz-Ruiz, Rebeca del Prado-Álvarez, Carlos Romero-Morales, María José Díaz-Arribas

**Affiliations:** 1 Faculty of Sport Sciences, Universidad Europea de Madrid, Calle Tajo s/n, 28670 Villaviciosa de Odón, Madrid, Spain; 2 Department of Radiology, Rehabilitation and Physiotherapy, Faculty of Nursing, Complutense University of Madrid, Plaza Ramón y Cajal nº 3, Ciudad Universitaria, 28040 Madrid, Spain

**Keywords:** Health care, Urology

## Abstract

In the last decade, minimalist shoes have gained popularity as an alternative to traditional shoes. The aim of the present study was to determine the short-term effects of minimalist shoes in femur range of motion (ROM) and cadence. The secondary objectives were the assessment of the electromyographic activity of the pelvic floor muscles (PFM) in nulliparous women. A randomized, prospective cross-over clinical trial design was used for the study. A total of 51 participants were randomly allocated into a two-sequence crossover design (AB/BA crossover design). Femur ROM, cadence and PFM activity were recorded. The femur ROM at 6 km/h was greater with the minimalist shoes by 1.62 degrees than with the traditional ones (*p* = 0.001). There was a main effect of the type of shoe (*p* = 0.015) systematically observing a higher running cadence with the minimalist shoe compared to the traditional one. Electromyographic activity of the PFM revealed significant differences for 11 km/h for the total average (*p* = 0.027) and the minimum peaks at 9 km/h (*p* = 0.011) and 11 km/h (*p* = 0.048) for the minimalist shoe with respect to the traditional shoes. Minimalist shoes produce immediate effects on the biomechanical variables of running. An increase was observed in the femur ROM at 6 km/h and in the cadence at 11 km/h with the use of minimalist shoes. The use of minimalist shoes increased the electromyographic activation of the PFM in the minimum peaks at speeds of 9 and 11 km/h and in the total average at speeds of 11 km/h compared to the traditional shoe.

## Introduction

Over the last 50 years, running shoes have undergone an immense change, since the appearance of the minimalist footwear used by our ancestors^1^ to nowadays, with the boom in cushioned shoes, new materials, elevated heels, corrective devices and technological materials^2^.

However, in the last decade, minimalist shoes have gained popularity as an alternative to traditional shoes. Minimalist footwear was developed by running shoe companies in response to the "barefoot movement"^3^, which advocates natural running which humans have adapted naturally over millions of years of evolution. This trend is gaining strength as an alternative to specialized sport shoes because it has not been possible to reduce the incidence of injuries, with 68.3% of runners reported to have had an injury in the previous year. Besides, 81.45% of these injuries is believed to be running related^4^.

Followers of the minimalist side support the opinion that it is more efficient and reduces injury risk^5^ due to modification through forefoot strike^6^, increasing stride cadence, decreasing active peak vertical force^7^ and modifying kinematics and other factors of the lower limbs^8^, aiming as a mean goal that the human body actively organizes itself to decrease load rate. On the contrary, opponents argue that the foot needs to be protected by the stability, cushioning and support that high-tech devices provide, specific to traditional shoes^9^, in order to improve comfort, safety, performance and running economy.

Therefore, reducing injuries and improving performance through the use of running shoes have become a major issue in both the sport industry and investigation^10^. Numerous studies compare the different types of footwear without reaching any consensus^11–13^.

Previous studies apply the electromyography (EMG) method to analyze the lower limb muscles^14^ and evaluate the effect of minimalist shoes^15,16^; nevertheless, no prior research has studied the activity of the PFM during running using different footwear, despite the high prevalence of stress urinary incontinence derived from high impact exercise in nulliparous women^17^.

The hypothesis of the present study is that the use of minimalist footwear produces biomechanical changes and an increase in the activation of the PFM. The aim of the present study was to determine the short-term effects of minimalist shoes in femur ROM and cadence compared to traditional shoes during running. The secondary objectives were the assessment and comparison of the EMG activity of the PFM in nulliparous women at three different velocities (6, 9, 11 km/h) during running with the use of two types of shoes: minimalist and traditional.

## Materials and methods

### Study design

A randomized, prospective cross-over clinical trial design was used for the study. A total of 51 participants were randomly allocated into two groups according to the order of use of the running shoes, having a two-sequence crossover design (2 × 2 or AB/BA crossover design). The intervention A used minimalist shoes, the intervention B used traditional shoes. The randomization of sequence of the footwear was based on the table of random permutations by Moses and Oakford^18^. It complies with the guidelines prescribed by the CONSORT checklist.

### Ethical considerations

The study was approved by the local Research and Ethics Committee of Hospital Clinical San Carlos, (code 19/570-E_TFM) which complied with all the principles set forth in the Declaration of Helsinki. All participants signed informed written consent forms to participate in this study. This trial was also registered in clincaltrials.gov (CI: NCT04457141).

### Participants

Participants were recruited from the Complutense University in Madrid. A total sample of 51 nulliparous women (to avoid dysfunctions derived in the PFM from pregnancy and childbirth) were included in the study and met all of the following criteria: they were aged between 18 and 38 years, were clinically healthy, physically able to run on a treadmill, had a BMI less than 30 kg/m^2^, and used traditional shoes in their sport practice.

The exclusion criteria were pregnant women, autoimmune illness, lower limb surgery in the previous 6 months, neurologic disorders, and inability to run for 90 s.

### Sample size calculation

The selection of the sample size was determined by convenience based on the only previous study on the evaluation of the PFM during running at different speeds carried out by Koenig et al.^21^ with a sample of 50 participants. According to a possible 10% loss to follow-up, a total sample size of 51 participants was recruited.

### Type of footwear

**Figure 1:**
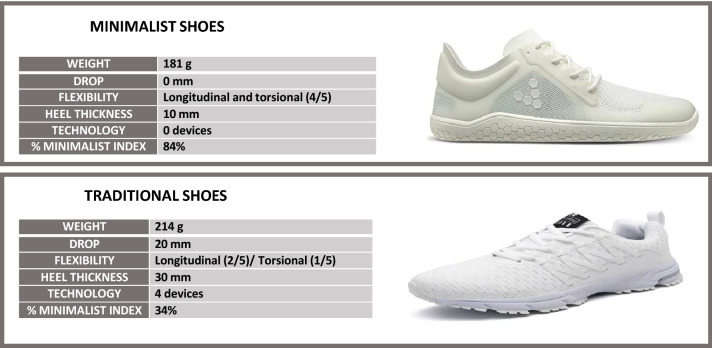
Minimalist and traditional shoe description.

Vivobarefoot Primus Lite III (Vivobarefoot, London, UK) was used as a minimalist shoe by all the participants and Sollomensi (Sollomensi, China), was used as a traditional shoe. The footwear was provided by the brands exclusively for the study and all participants used the same footwear models during the study. Figure 1 describes the characteristics of the two types of footwear that were used: weight, thickness of the sole, drop, length and torsional flexibility technology device. These characteristics determine the percentage of the minimalist index (MI) of each shoe, as established in the Delphi study carried out by Esculier et al. in which they created the MI scale^22^.

### Procedure

Age, weight, body mass index, health, and daily physical activity information were recorded. Regarding the EMG evaluation, an intracavitary EMG periform probe™ (Neen, HealthCare, Dereham, United Kingdom) was used to collect PFM data, this probe features a device cable attached to the proximal 1/3 of the anterior femoral area and integrated long leads for connection to the EMG. The "Periform" intravaginal probe has a unique rectangular section that resists lateral movements to prevent unwanted movements and the oval shape of the electrode provides a comfortable self-application. This shape creates a vacuum effect to prevent dislodgement, especially in nulliparous women. Besides, a ground electrode and amplifier was placed on the right iliac crest to reduce noise in accordance with the SENIAM recommendations^23^. Following these recommendations, skin preparation and electrode placement were also performed, using the musculoskeletal models for sEMG, in which the muscle is conceived as a large muscle fiber, monitoring the average properties of the whole muscle to avoid localized effects of independent motor units^23^. The EMG system used was a mDurance^®^ (211) (mDurance Solutions SL, Granada, Spain) for the analysis of the sEMG activity of the PFM. Accelerometers (Shimmer3 Consensys IMU,Dublin, Ireland) were attached at the proximal third of the rectus femoris to objectively record space-temporal parameters of the running cycle measuring the path of the femur from the maximum point of flexion to the maximum point of extension, thus calculating the total flexo-extension movement of the hip. EMG activity was recorded in the standing position for 2", serving as a reference point for the analysis. Maximal voluntary isometric contraction (MVIC) for the PFM was measured in order to normalize the electromyographic signal during the 3 times 10 s with 20 s of rest between each contraction. This test was performed in a supine position with the knees flexed at 90 degrees.

Finally, the subjects walked on a treadmill HP Cosmos, model Mercury (Ref.cos 30000va08, Hp/cosmos Sport and Medical, Nussdorf-Traunstein, Germany). The activity was recorded 30" for each speed (6, 9, 11 km/h), for 5 min to warm-up ran at low intensity (less 6 km/h) at a free velocity; and then EMG and accelerometers were measured while the women ran for 30 s at 6 km/h, 30 s at 9 km/h and 30 s at 11 km/h under different footwear conditions: minimalist and traditional shoes with a wash time of 5 min between each intervention, in which subjects remained seated, rested and changed their shoes to repeat the same protocol with the other shoes. Once the electromyographic activity of the SP was recorded during the 90" of running on the treadmill, the signal was first normalized with the MVIC monitored at the beginning of the session and then the electromyographic data was analyzed in detail to obtain the following variables: the total average, the average of the minimum peaks and the average of the peaks.

### Statistical analysis

SPSS software (IBM, Armonk, NY, USA) and Jamovi (Jamovi 2.0) were employed for the statistical analysis. The Kolmogorov–Smirnov test was used to check the normality assumption of each variable. A descriptive analysis was carried out with the mean and SD for each variable in both groups. Repeated-measures analysis of variance (ANOVA) with 2 factors (considering the significance of the Greenhouse–Geisser correction when the Mauchly test rejected the sphericity) and the Bonferroni correction were applied to determine the intergroup comparison for two biomechanical variables such as ROM and cadence and, the EMG variable to evaluate the activity in the PFM (2 groups: minimalist and traditional group × 3 measurements: 6, 9 and 11 km/h). In addition, the partial eta squared coefficient (η2) was employed for the effect size calculation. For the effect sizes interpretation values of 0.01, 0.06 and 0.14 for small, medium and large effects were considered, respectively^24^. In cases where there was asymmetry of the distributions with outliers, a logarithmic transformation (LN) of the data was performed with the SPSS program, taking these transformed scores as a reference for the analysis. All the statistical tests were performed with a 95% confidence interval (*p* < 0.05).

## Results

### Baseline demographic analysis

The sample included in the study was of 51 participants. Table 1 shows the demographic characteristics of the total sample. (Table 1) A large intersubject variability can be observed in the descriptive data on the sample, with large standard deviations in some characteristics, especially in weight and age. The demographic characteristics of the sample are heterogeneous.

**Table 1:** Sociodemographic data of the sample.

	Mean ± SD
Age	26.55 ± 5.11
Weight	58.24 ± 7.06
Height	1.65 ± 0.06
BMI	21.29 ± 2.07

*SD* standard deviation.

### Biomechanical variables

#### Femur ROM

The descriptive statistics of the femur ROM can be consulted in Table 2. The variations of the scores were due in 93% to the speed factor, F (1.25, 21,912.82) = 703.79, *p* < 0.001, ηp2 = 0.93, finding significant differences in all the comparisons between the three speeds. No main effect of shoes on femur ROM was found, F(1,50) = 1.95, *p* = 0.169, ηp2 = 0.04. However, the pairwise analysis revealed differences between the shoes at the speed of 6 km/h (Table 2). The range of the femur at 6 km/h was greater with the minimalist shoes by 1.62 degrees than with the traditional ones (Table 2).

**Table 2:** Effects of shoes in biomechanical variables.

Measure	Minimalist shoes (MS)	Traditional shoes (TS)	Pairwise comparisons			Time value
			Differences (MS-TS)	IC 95%	p_bonferroni_	F (Df); *p* (Eta^2^)
**Fémur ROM**						F(1,50) = 1.95, *p* = 0.169, η_p_^2^ = 0.04
6 km/h	38.13 ± 4.22	36.51 ± 4.28	1.62	0.77–2.47	< 0.001*	
9 km/h	50.16 ± 6.35	49.44 ± 5.80	0.73	− 0.63–2.08	0.285	
11 km/h	57.95 ± 7.86	57.84 ± 7.41	0.11	− 1.64–1.85	0.904	
**Cadence**						F(1,49) = 6.42, *p* = 0.015, η_p_^2^ = 0.12†
6 km/h	74.23 ± 4.06	73.87 ± 4.04	0.41	− 0.32–1.14	0.261	
9 km/h	79.88 ± 4.40	79.53 ± 4.05	0.42	− 0.22–1.06	0.196	
11 km/h	84.01 ± 5.14	82.82 ± 5.06	1.20	0.36–2.04	0.006*	

*Bonferroni post-hoc test was used, †Greenhouse–Geisser correction was used.

Significant values are in bold.

#### Cadence

**Figure 2:**
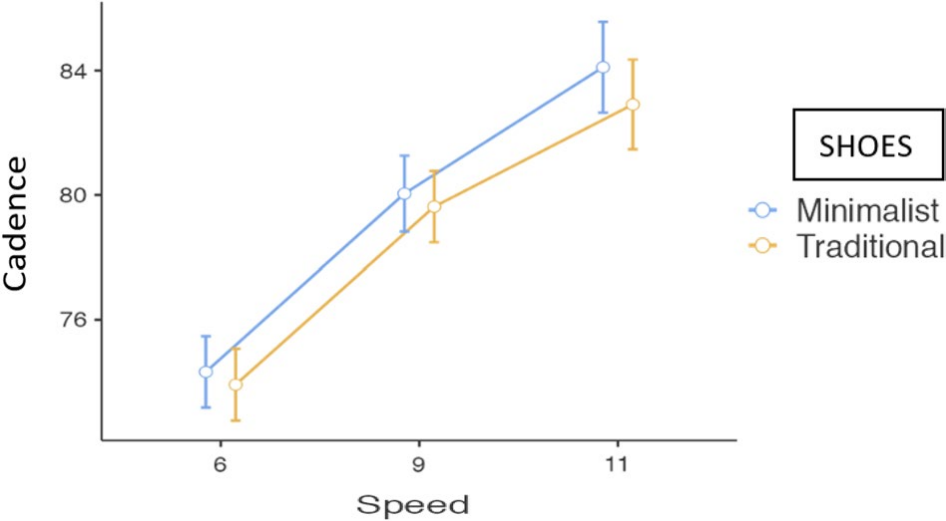
Estimated marginal mean of cadence scores by speeds and by shoes. Error bars represent the standard error of the mean.

Running cadence ranged from 74 to 82 steps per minute (Table 2). The ANOVA found a large effect of speed on cadence, F(1.22,59.99) = 268.40, *p* < 0.001, ηp = 0.85.The ANOVA also found a main effect of the type of shoe, F(1,49) = 6.42, *p* = 0.015, ηp2 = 0.12, systematically observing a higher running cadence with the minimalist shoe compared to the traditional one (Fig. 2). Subsequent pairwise analysis revealed significant differences in cadence only at the speed of 11 km/h (Table 2). With the minimalist shoe, the women took 1.20 steps more than with the traditional shoe. No interactions were found between speed and shoes, leaving the simple interaction effect closest to significance in the comparison of shoes between speeds 9 and 11 km/h, *p* = 0.073. (Table 2) (Fig. 2).

#### Electromyography variables

Three variables of the electromyographic data were studied: the average of the maximum peaks recorded, the average of the minimum peaks, and the total average of the EMG trace. The exploratory and descriptive analysis revealed a great variability in the distribution of these 3 variables. In the descriptive statistics of the 3 electromyographic variables, the difference between the mean and the median, the wide confidence intervals, or the high standard deviation can be consulted (Table 3).

**Table 3:** Effects of shoes in electromyographic variables.

Measure	Difference (MS-TS)	IC (95%)	p_bonferroni_
**Peak max**			
6 km/h	0.08	− 0.01–0.18	0.088
9 km/h	0.06	− 0.02–0.14	0.111
11 km/h	0.06	0.01–0.13	0.057
**Peak min**			
6 km/h	0.03	− 0.01–0.07	0.096
9 km/h	**0.05**	**0.01–0.09**	0.011*
11 km/h	**0.04**	**0.00–0.08**	0.048*
**Total average**			
6 km/h	0.06	0.00–0.12	0.058
9 km/h	0.04	− 0.01–0.08	0.092
11 km/h	**0.05**	**0.01–0.09**	0.027*

*Bonferroni post-hoc test was used.

Significant values are in bold.

The asymmetry analysis of the sample distributions presented abnormally positive values of up to 7.24 points. In these cases, the right tails extended to very high values. For this reason, we decided to carry out a logarithmic transformation (LN) of the data with the SPSS program. After this transformation, the histograms presented normalized shapes, with a maximum asymmetry value of 1.86 –within acceptable margins. Therefore, MR ANOVAs were carried out taking these transformed scores as reference.

##### Maximum peaks

The ANOVA also found large differences in the maximum peaks as a function of speed, the higher the speed, the greater the activation. EMG activation with the minimalist shoe was consistently higher than with the traditional shoe. However, despite this continued behavior, the difference was very small, far from being significant. Thus, ANOVA ruled out any effect (neither main, nor interaction effects) of shoe type on EMG peaks. The subsequent post-hoc analysis also revealed no difference between traditional and minimalist shoes and speed (Table 3).

##### Minimum peaks

The post-hoc analysis revealed significant differences between shoes. The minimalist shoe did reflect a greater activation than the traditional shoe in the PFM at the speed of 9 km/h and 11 km/h (Table 3).

##### Total average

**Figure 3:**
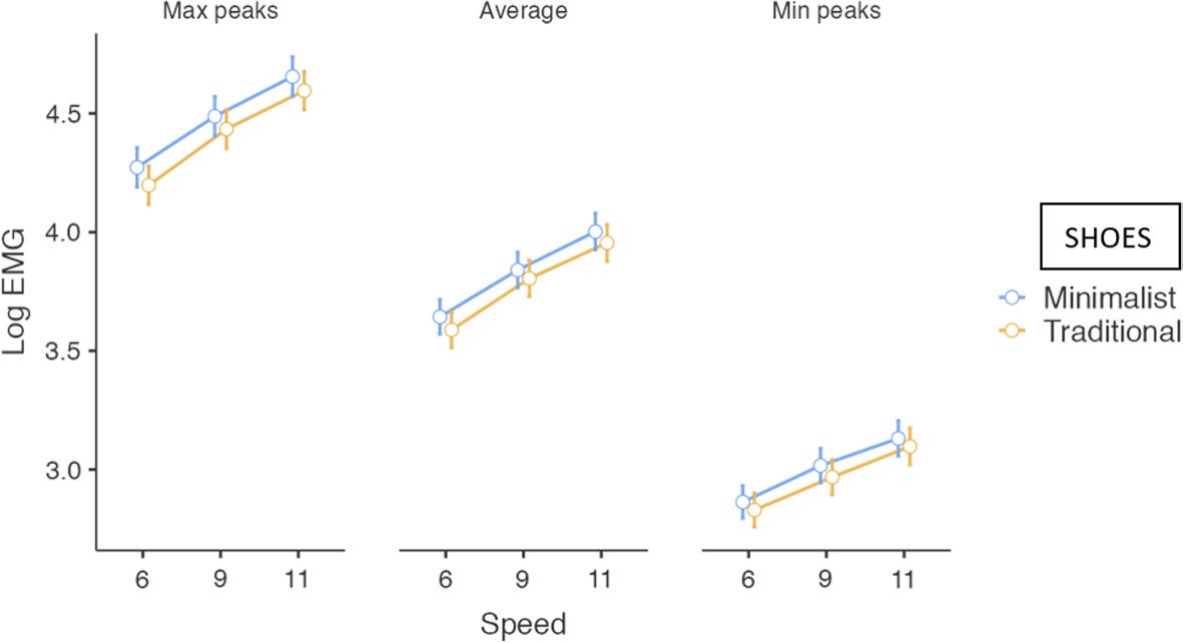
Estimated marginal mean of electromyographic activity scores by speeds and by shoes in PFM. Error bars represent the standard error of the mean.

As was the case with the previous EMG variables, it was observed that electromyographic activation was slightly higher with the minimalist shoe compared to the traditional shoe. Despite this repetitive trend, the effect of shoe type was not significant overall. On the other hand, the post-hoc analyses in which the contrasts between shoes are described in detail, corroborated that the minimalist shoes induced greater total electromyographic activation in the PFM at the speed of 11 km/h (Table 3) (Fig. 3).

## Discussion

The results of the present study concluded that during running with minimalist shoes, there is an increase in the femur ROM at 6 km/h in the sagittal plane and an increase in cadence, compared to traditional footwear, which translates into variations in running technique.

With respect to the EMG results, there is an increase in electromyographic activity of the PFM with minimalist shoes.

During running with minimalist shoes there was increased hip ROM in the sagittal plane at 6 km/h^25^, that may be due to a greater activation of the surrounding muscles due to an increase in sensory information with minimalist shoes. In addition, the use of this footwear produces a modification of the tread pattern towards this increased support in the forefoot^26^ and changes in the angles of the knee^27^ and the hip^25^ during the movement.

A change of footwear was made without a previous transition to minimalism. Therefore, the results obtained could differ from those of runners who usually wear minimalist shoes^28^. The lack of concordance between these studies and our results could be due to the fact that the cohorts chosen in these studies were highly trained men runners.

A systematic increase in cadence when the runners used the minimalist shoes compared to the traditional shoes was found in our study, and significant differences were revealed at high speeds (11 km/h).

Increasing the cadence raises the time of the flight phase and decreases the support time. In this way, the total impact forces are reduced and a more uniform load distribution^29^ is achieved, which is especially necessary when running fast, since the higher the speed, the more impact. It also produces energy absorption of impact forces in lower limb joints while running. Impact forces are associated with injuries such as patellofemoral pain syndrome^30^ and iliotibial band syndrome^31^ with a higher incidence in women runners compared to men^32^.

The EMG activation in this study was collected through 3 variables: total average, minimum peaks and maximum peaks. Statistically significant differences were found in the total average at high speeds (11 km/h), as well as in the minimum peaks of electrical activation of the PFM both at speeds of 9 km/h and 11 km/h; in both cases obtaining higher values with the use of minimalist footwear compared to traditional shoes.

Higher muscle activation is related to increases in joint stability and improvements in urinary continence capacity by increasing the absorption of the impact derived from running in the PFM^33^. Likewise, in running at fast speeds, the highest activation values are associated with a reflex activity by the PFM as a preventive mechanism of the impact^34^. In addition, it has been theorized that greater activation is an active mechanism that the body uses as a buffer^35^, that it is associated with structural changes such as an increase in the cross section of the muscle^36^, and may be a potential aid for prevention and treatment of injuries in runners.

It could be thought that if with the measurements carried out in a short period of time as in this study, differences in muscle activation have already been found, it could be the case that after a period of training, more powerful results would be achieved in all the musculature tested.

### Clinical applications

The use of minimalist footwear can be a modifying factor of the running technique in nulliparous women runners, directing the biomechanics of this running to protective parameters against musculoskeletal injuries of the lower limbs. Besides, minimalist shoes compared to traditional ones can be a preventive factor for PFM dysfunctions, thanks to the increase in activation during running with minimalist shoes.

Traditional shoes do not produce greater changes compared to minimalist shoes in biomechanical and electromyographic variables compared to minimalist shoes, even with the technological advances of cushioning and support.

### Methodological considerations

This study was not single blinded. Neither the therapist who carried out the measurements, nor the patient, could have been blinded to the different characteristics of both shoes which are easily recognizable. Only the therapist who evaluated the data obtained was blinded.

Discomfort that the use of the intravaginal EMG probe could entail for novice runners was taken into account, but the benefit of obtaining these variables was weighed over the slight discomfort of using the internal probe.

Finally, the running speeds were chosen externally, with the runners having to adapt to these speeds, so that a free run was not carried out, which could lead to involuntary changes in the running style.

### Future studies

New lines of research are opened up, especially to study the effect of minimalist footwear in women with stress urinary incontinence (SUI).

## Conclusion

Minimalist shoes produce immediate effects on the biomechanical variables of running, finding an increase in the femur ROM at 6 km/h and in the cadence at 11 km/h with the use of minimalist shoes. An exploratory and descriptive analysis of the electrical activation of the PFM during running revealed a great variability in the distribution of the total average, of the minimum peaks and of the maximum peaks. In addition, it was observed that the use of minimalist shoes increased the electromyographic activation of the PFM in the minimum peaks at speeds of 9 and 11 km/h and of the total average at speeds of 11 km/h compared to the traditional shoe.

## Data Availability

The datasets used and/or analysed during the current study are available from the corresponding author on reasonable request.
